# Rapamycin extends life- and health span because it slows aging

**DOI:** 10.18632/aging.100591

**Published:** 2013-08-11

**Authors:** Mikhail V. Blagosklonny

**Affiliations:** Department of Cell Stress Biology, Roswell Park Cancer Institute, BLSC, L3-312, Elm and Carlton Streets, Buffalo, NY, 14263, USA

**Keywords:** murine lifespan, mammalian target of rapamycin, rapalogs, diseases, age-relayed pathology

## Abstract

Making headlines, a thought-provocative paper by Neff, Ehninger and coworkers claims that rapamycin extends life span but has limited effects on aging. How is that possibly possible? And what is aging if not an increase of the probability of death with age. I discuss that the JCI paper actually shows that rapamycin slows aging and also extends lifespan regardless of its direct anti-cancer activities. Aging is, in part, MTOR-driven: a purposeless continuation of developmental growth. Rapamycin affects the same processes in young and old animals: young animals' traits and phenotypes, which continuations become hyperfunctional, harmful and lethal later in life.

Found by chance on the mystical Easter island [1], the anti-aging drug rapamycin gave birth to numerous myths. This time, it is claimed that rapamycin prolongs lifespan and prevents aging-associated changes by aging-independent mechanisms, not by affecting aging itself [2]. But what is then aging itself.

## What is aging?

Aging is an exponential increase of the probability of death with age [3]. No one has died from health or without a cause. Most elderly humans die from age-related diseases [4-10], which are also called “natural causes”, if a precise diagnosis is unnecessary. In mammals, death from aging means death from age-related diseases. Not only humans and other mammals but also aging worms and flies die from pathologies [11-27].

## Diseases are biomarkers of aging

Age-related diseases are biomarkers of aging [8]. The most common are cardiovascular diseases (associated with atherosclerosis, hypertension and cardiac hypertrophy), cancer, diabetes (and other complications of metabolic syndrome), Alzheimer and Parkinson diseases, macular degeneration and so on. Many manifestations of aging are not considered as diseases because they develop in everyone (e.g. female menopause).

The distinction is arbitrarily. For example, cancer-prone transgenic mice can exclusively die from cancer but still cancer is a disease. But many alterations, although associated with age, are not involved in aging. And these phenotypes are not affected by rapamycin.

## Cellular aging

Unless we believe in vitalism, organismal aging should be linked to cellular aging. Cellular aging is, in part, MTOR-dependent process. The MTOR (mechanistic or mammalian target of rapamycin) pathway is activated by growth factors, hormones (such as insulin and testosterone), nutrients, oxygen and some conditions such as obesity [28-38]. Figuratively, MTOR is a “molecular hypothalamus”, a sensing pathway in every cell [39]. In turn, MTOR stimulates specific functions of differentiated cells and cellular mass growth. In proliferating cells, growth is balanced by division. In resting cells, active MTOR causes cellular hypertrophy, hyperfunctions (such as hypersecretion). MTOR-driven geroconversion is a conversion from quiescence to senescence [40-59]. And cellular senescence is characterized by increased cell-type-specific cellular functions (hyperfunctions), altering homeostasis and leading to age-related diseases [9].

## Systemic hyperfunctions and aging

Except of terminal stages of age-related diseases, aging is associated with systemic hyperfunctions: increased blood pressure (hypertension), increased platelet aggregation (hyper-aggregation), hyper-contractility of arterial smooth muscle cells, hyper-coagulation, hyperlipidemia, hyperglycemia, hyperinsulinemia, increased resistance to hormones, pro-inflammatory conditions, organ hypertrophy, fibrosis and hyperplasia. These hyperfunctions are damaging to the organs and, when damage occurs, then some functions are lost. So only late stages of aging are decline and loss of functions. Terminal stages are MTOR-independent and will not be reversed by rapamycin. For example, hyperfunctional osteoclasts cause osteoporosis, leading to a broken bone and a sequence of events (immobilization, pneumonia, etc), which require standard medical interventions, not anti-aging drugs [60]. Not only in mammals, but also in *C elegans* and *Drosophila*, life-limiting pathologies are caused of exacerbated and intensified normal processes and functions [19,25,61,62].

**Aging processes do not spring from nothing. They are continuations of normal cellular, tissue, organ and system functions in young animals. Unless miracle is possible, rapamycin must affect the same processes in old and young animals. And it does**.

## Aging is a quasi-program (not a program)

Why systemic hyperfunctions arise? Aging is an unintended continuation of organismal growth, like cellular senescence is a continuation of cellular growth [63]. In other words, aging is a quasi-program (not a program): an unintended and purposeless continuation of developmental programs, which are not switched off upon their completion [64-67], causing age-related diseases. For example, blood pressure is increased from birth to adulthood and continuation of this trend leads to hypertension. Menopause is a hyperfunctional continuation of reproductive program [68]. Aging-associated pathologies are continuation of normal functions of the young organism. Therefore, rapamycin must affect the same processes in young and old animals, because aging is a continuation of normal functions. Aging processes do not spring from nothing. They are continuations of normal cellular, tissue, organ and system functions in young animals.

**Rapamycin extends life span independently of its anti-cancer effect and prevents cancer by slowing down aging**.

## Cancer and aging

Cancer is an aging-related disease and interventions that slow aging (e.g. calorie restriction) delay cancer [69-78]. Furthermore, compared with calorie restriction, rapamycin stronger inhibits MTOR. It is predictable that if rapamycin slows aging, it should delay cancer [79,80]. Studies support these predictions [81-84] and rapamycin extended lifespan and delayed cancer, even when calorie restriction did not [85]. Although rapamycin is a potent cancer-preventive agent, it is only modestly effective for cancer treatment. Rapalogs are most effective in drug combinations [86-93]. They also may decrease side effects by suppressing senescence of normal cells [51,58,59,94,95]. Also, senescence of normal cells creates cancer-promoting micro-environment [96-103]. If rapamycin indeed prevents cancer by slowing aging (not by killing cancer cells), the prevention must be started *before* cancer is initiated. In other words, if rapamycin treatment is started too late in life, then its anti-cancer effect will be blunted. This was shown in cancer-prone p53+/− mice [104]. The same was shown by Neff et al: rapamycin rapamycin did not prevent cancer when the treatment was started at middle and old age [2]. Thus, the JCI study confirms the notion that rapamycin delays cancer by slowing aging (see also discussion here in the last section). Anti-cancer effects simply cannot be responsible for life extension by rapamycin. First, effective anti-cancer drugs that are curative in lymphomas, testicular and ovarian cancers (methotrexate, cisplatin, paclitaxel) would greatly shorten murine lifespan, especially when started in young age. Even further, typical anti-cancer drugs accelerate cancer. For example, radiation (a classic anti-cancer intervention) dramatically accelerates cancer in p53+/− mice and shortens life span [105-109]. And anti-cancer drugs cause secondary cancers in patients. In contrast, not only rapamycin extends lifespan, it is the only known drug that extends life span consistently. Second, apart from cancer-prone strains of mice, cancer is not the main cause of death in most animals. MTOR is involved in most age-related diseases and rapamycin prevents them in mammals [64,110-123] and slows down aging [81,124-127]. Finally, yeast, worm and flies do not die from cancer and still inhibition of the MTOR pathway extends lifespan [128-137].

## Inhibition of TOR slows aging: converging evidence [124]

1. Rapamycin suppresses geroconversion: conversion from cellular quiescence to senescence. Geroconversion is cellular basis of organismal aging
2. Genetic manipulations that inhibit the TOR pathway extend life-span in diverse species from yeast to mammals
3. Rapamycin extends lifespan in all species tested
4. Calorie restriction, which inhibits MTOR, extends lifespan
5. MTOR is involved in diseases of aging and rapamycin prevents these diseases in animal models

## Rapamycin slows aging: the JCI paper [2]

How does the Neff et al study support the model of quasi-programmed aging?

1. As shown by Neff *et al*, chronic administration of rapamycin extends lifespan in male C57BL/6J mice, when started at both young and old age. Note: This extension is impressive given that (a) effects of rapamycin in male mice are blunted compared with female mice in previous studies, (b) C57BL/6J mice are intrinsically long-lived and (c) rapamycin was administrated in everyday schedule (chronic or immunosuppressive schedule) instead of intermittent or pulse administration (anti-aging schedule).
2. C57BL/6J mice are refractory to many tumors http://jaxmice.jax.org/strain/000664.html Therefore, life extension is difficult to explain by anti-cancer effects of rapamycin.
3. In fact, rapamycin did not prevent cancer when the treatment was started at middle and old age, but still extended life span. As stated by Neff *et al* [2]: “Rapamycin … had no measurable effect in the 25-month cohort (vehicle, 1 of 5; rapamycin, 2 of 8; P = 1.0, Fisher exact test) or the 34-month cohort (vehicle, 1 of 5; rapamycin, 3 of 10; P = 1.0, Fisher exact test).” As we discussed here, this indicates that effects of rapamycin are probably due to suppression of aging. Rapamycin treatment decreased cancer incidence only when it was started in young mice.
4. Rapamycin counteracted certain aging-related alterations in both young and old mice. This suggests that aging is a continuation of normal traits in young organisms. Aging is driven by intensified and exacerbated normal cellular functions.
5. Rapamycin did not affect many parameters that are not aging-specific such as alterations in plasma sodium, calcium and chloride concentrations. This is expectable. Aging is not associated with alterations of electrolyte homeostasis. These alterations are terminal phases of medical conditions due to organ (e.g. renal) failure.
6. Some age-related alterations actually counteract aging. For example, although RNA/protein synthesis is decreased with aging in model organisms, yet its further inhibition prolongs life span further [138-141]. As shown by Neff et al, rapamycin did not prevent *anti-aging* alterations such as a decrease in testosterone levels. Noteworthy, testosterone activates mTOR.
7. Some trends reported by Neff et al are not typical for aging. For example, while Neff reported a decrease in blood glucose and lipids with age, these parameters tend to increase with age, especially when age-related diseases develop. Perhaps mice with hyperglycemia and hyperlipidemia died during the study, while only surviving (the healthiest) mice were examined at the end of the study.
